# Unraveling the mechanisms underlying air pollution-induced dysfunction of the oral–gut–brain axis: implications for human health and well-being

**DOI:** 10.2478/abm-2025-0002

**Published:** 2025-02-28

**Authors:** Sisi Chen, Wenlei Yu, Yiwen Shen, Linjie Lu, Xiangyong Meng, Jun Liu

**Affiliations:** Department of Stomatology, Huzhou Wuxing District People's Hospital, Huzhou Wuxing District Maternal and Child Health Hospital, Huzhou, 313008, China; Department of Oncology, The Affiliated Cancer Hospital of Nanjing Medical University, Jiangsu Cancer Hospital, Jiangsu Institute of Cancer Research, Nanjing, 210009, China; Department of Stomatology, Haining Hospital of Traditional Chinese Medicine, Jiaxing, 314400, China; Department of Stomatology, Medical School, Huzhou University, Huzhou, 313000, China; Department of Stomatology, The First Affiliated Hospital of Huzhou University, Huzhou, 313099, China

**Keywords:** air pollution, health effects, inflammation, microorganisms, oral–gut–brain axis

## Abstract

Air pollution exposure has become an international health issue that poses many risks to life and health. The bidirectional regulatory network, known as the oral–gut–brain axis connects the oral cavity, intestine, and central nervous system, as well as its influence on health outcomes from exposure to air pollution is receiving increased attention. This article systematically details the epidemiological evidence linking air pollutants to diseases affecting the oral, respiratory, intestinal, and nervous systems, while also explaining the route of air pollutants via the oral–gut–brain axis. The oral–gut–brain axis anomalies resulting from air pollution and their underlying molecular processes are also covered. The study provides a fresh viewpoint on how exposure to air pollution affects health and investigates cutting-edge preventative and therapeutic techniques.

Air pollution has turned into an increasing healthcare issue worldwide. The Lancet's 2019 survey data revealed that worldwide air pollution provided in excess of compared with 3% of disability-adjusted life years (DALYs) and caused 2.92 (11.3%) and 3.75 million (12.2%) deaths in women and men, respectively [1]. Various air pollutants significantly affect health, as research has shown that airborne contaminants can damage multiple organs and systems, including the respiratory, cardiovascular, and nervous systems [2]. Additionally, studies on disease transmission have demonstrated a robust link within air pollution as well as several chronic conditions like heart and brain diseases, respiratory issues, diabetes, memory loss, Parkinson's disease, and other long-term health complications, along with an increase in mortality rates [3,4,5,6].

Within the past decades, the idea of the in vivo oral–gut–brain axis has gained prominence in life sciences and health disciplines, describing the complex bidirectional network of exchanges involving the oral cavity, the intestines, and the central nervous system. This network involves multiple regulatory systems, including the nervous, endocrine, and immune systems [7, 8]. The oral cavity and the intestines are critical portals for the body's exchanges alongside the external environment, and the health of the oral and intestinal flora and mucosal barriers is crucial. The oral and gut microbiota share similar functions in maintaining host health by regulating immune responses, metabolizing nutrients, and preventing pathogen colonization [9, 10]. These microbial communities engage in complex cross-talk with the host's immune system, modulating the balance between pro-inflammatory and anti-inflammatory responses, which is essential for maintaining homeostasis and preventing chronic diseases [11, 12]. Moreover, the oral and intestinal mucosal barriers, composed of epithelial cells connected by tight junctions, play a critical role in selectively permitting the passage of nutrients and immune cells while restricting pathogen invasion, thus acting as the first line of defense against external insults [13, 14]. Disruptions in the connection within the mouth, gut, and brain are strongly associated with various systemic diseases like obesity, diabetes, heart conditions, and neurodegenerative disorders [15,16,17]. As multiple investigations have demonstrated, breathing in air pollution may throw off the equilibrium of microorganisms in the body and damage the protective linings of the gut and respiratory systems. This disruption can extend to the central nervous system through the interconnected network of the mouth, gut, and brain, potentially leading to various health issues [18,19,20].

This paper will comprehensively discuss air pollution's effects upon the oral–gut–brain axis and its underlying mechanisms, presenting a new perspective for exploring the health risks connected to air pollution and identifying potential intervention strategies for diseases connected to air pollution.

## Composition and health effects of air pollutants

Air pollutants comprise a complex mix of harmful materials, which include particulate matter (PM), gaseous pollutants, volatile organic compounds, as well as heavy metals. These pollutants can damage various organ and body systems, potentially leading to premature death. Understanding the composition of air pollution elements and their health impacts is necessary to create efficient control measures and safeguard public health **(Table 1)**.

**Table 1.** Composition and health impacts of major air pollutants

**Air pollutant**	**Sources**	**Health effects**	**Reference**
PM2.5	Complex mix of solid and liquid particles	Mouth, intestinal, and nervous system disorders; increased risk of various diseases	[21, 29]
O_3_	Highly reactive oxidizing gas	Chronic inflammation; neurodegenerative and intestinal conditions	[31, 32]
SO_2_	Burning fuels containing sulfide	Respiratory issues; increased risk of mortality, dental problems, enteritis, and stroke	[33, 36]
NO_2_	Burning fossil fuels and vehicle emissions	Mucous membrane damage; increased risk of peptic ulcer bleeding and Parkinson's disease	[39, 42]
CO	Incomplete combustion of hydrocarbons	Tissue oxygen deprivation; increased risk of oral conditions, Sjogren's syndrome, and migraine	[43, 46]
PAHs	Incomplete combustion of organic matter	Toxic, mutagenic, and carcinogenic; increased risk of oral cancer, brain shrinkage, and motor impairments	[48, 58]
Dioxins	Waste burning, chemical production, steel plant operations, vehicle use, and accidental landfill fires	Accumulate in living organisms; increased risk of dental problems, inflammatory bowel conditions, and cognitive decline	[49, 53, 56]
Heavy metals (Chromium, Pb, Hg, Cd)	Various industrial and anthropogenic activities	Renal, cardiovascular, and neurological issues; increased risk of diabetes and cancer	[57, 59]

PAHs, polycyclic aromatic hydrocarbons.

A mixture of solid and liquid particles floating in the atmosphere is called PM, categorized via size as PM10 (≤10 μm), PM2.5 (≤2.5 μm), along with ultrafine particles (≤0.1 μm) based on aerodynamic diameter [21]. PM2.5, with its substantial surface area, attracts a larger number of hazardous substances and can penetrate the alveoli, increasing health risks [22]. Research has also found that PM2.5 particles carry various toxic substances on their surface. These particles can penetrate the skin through hair follicles, enter the body via inhalation through the mouth or nose, or be ingested with contaminated food. Once in the bloodstream, PM2.5 can be distributed throughout the body, affecting multiple organs and systems [23, 24]. Exposure to PM2.5 is strongly connected to disorders affecting the mouth, the intestines, and the nervous system. Studies show that for every 10 μg/m^3^ rise within PM2.5 levels, there is about a 10% rise within the likelihood of elevated high-sensitivity C-reactive protein (hs-CRP) within individuals alongside periodontitis [25]. Taiwanese men exposed to high levels of PM2.5 have a higher incidence of oral cancer [26]. Furthermore, children with greater exposure to PM2.5 have an increased risk of inflammatory bowel disease [27]. Additionally, each 10 μg/m^3^ increase in PM2.5 exposure significantly raises the risk of developing Parkinson's disease [28]. Other research revealed that a 4.34 μg/m^3^ rise within PM2.5 exposure increased the likelihood of developing Alzheimer's disease (AD) by 138%, as reported by Jung et al. [29] in 2015.

The primary gaseous pollutants include ozone (O_3_), sulfur dioxide (SO_2_), nitrogen dioxide (NO_2_), and carbon monoxide (CO) [30]. O_3_ is a highly reactive oxidizing gas linked to adverse health outcomes [31]. Investigations indicate that prolonged exposure to O_3_ may result in chronic inflammation within the body, which may contribute to neurodegenerative and intestinal conditions like AD, and inflammatory bowel and Crohn's disease [32]. SO_2_ is mainly produced by burning fuels containing sulfide, and inhalation can irritate and constrict the airways, increasing the risk of daily mortality [33]. Long-term exposure to SO_2_ has been connected to risks of under-mineralization of molar incisors [34], enteritis [35], and ischemic stroke [36] in children. Additionally, research has connected prolonged maternal exposure to O_3_ and SO_2_ pollution with abnormal oral facial fissure in infants [37, 38]. NO_2_, primarily emitted from burning fossil fuels and vehicle emissions, can damage the mucous membranes upon prolonged exposure [39, 40]. Long-term contact with NO_2_ has been displayed to increase the likelihood of peptic ulcer bleeding [41] and Parkinson's disease [42]. CO, a toxic gas produced from incomplete combustion of hydrocarbons, combines with hemoglobin to form carboxyhemoglobin, which leads to tissue oxygen deprivation [43]. Exposure to CO has been connected to an increased likelihood of developing potentially cancerous oral conditions [44], Sjogren's syndrome [45], and migraine [46].

Two common persistent organic pollutants (POPs) include dioxins and polycyclic aromatic hydrocarbons (PAHs) [47]. PAHs, primarily resulting through the incomplete combustion of organic matter like coal and tar, are toxic, mutagenic, and carcinogenic [48]. Dioxins often originate from activities such as waste burning, chemical production, steel plant operations, vehicle use, and accidental landfill fires. Known for their durability, dioxins accumulate in living organisms and can travel long distances, posing significant health risks [49]. Research has connected exposure to PAHs alongside an increased risk of developing oral cancer [50] and brain shrinkage [51]. Additionally, PAHs in air pollutants have exacerbated motor impairments following ischemic strokes by intensifying neuroinflammation [52]. Exposure to dioxin has been connected to a greater likelihood of developing enamel defects during tooth development and the loss of permanent teeth [53], discoloration in the mouth [54], inflammatory bowel conditions [55], and reduced cognitive function [56].

Prolonged contact with toxic heavy metals like chromium, lead (Pb), mercury (Hg), and cadmium (Cd) [57] may result in a number of health concerns, such as renal troubles, heart disease, nerve damage, and a greater likelihood of diabetes and cancer, contributing to high global illness and mortality rates [58]. Extended exposure to lead can accumulate in body parts such as the bones, brain, liver, and kidneys, resulting in neurological damage in infants and cardiovascular and renal issues in adults [59].

## The pathway through which air pollution components travel in the oral–gut–brain axis

The mouth is one of the first parts of the body exposed to air pollutants. These pollutants can directly deposit on the oral mucosal surface, destroy the barrier function of the oral mucosa, and cause oral inflammation [60, 61]. Research has found that PM2.5 and PM10 particles can directly deposit on the oral mucosa, causing genotoxic and cytotoxic damage to the epithelial cells of the oral mucosa [62, 63]. Infections in the mouth can extend to the lower respiratory and digestive systems through the “oral–lung axis” and “oral–gut axis,” exacerbating inflammation in the respiratory system, lung conditions, and intestinal issues [64,65,66]. Oral inflammation can also impact brain function via the “oral–brain axis,” particularly through the “oral–olfactory bulb–brain axis.” Toxins in the air and inflammatory agents in the mouth can travel through the olfactory epithelium, reach the olfactory bulb, and then spread to the brain, causing inflammation and degeneration of nerve cells [67,68,69,70]. Studies have shown that cerebrospinal fluid and hippocampal β-amyloid levels are increased in patients with periodontitis, indicating that oral inflammation can exacerbate the neuropathological changes associated with AD through the oral–brain axis [71,72,73]. Additionally, research has demonstrated that specialized dental care significantly improves the well-being of patients in the subacute stage of neurosurgical conditions [74].

Air pollutants are primarily absorbed through the respiratory system. Inhalation of fine particles such as PM2.5 can impact every respiratory system component, leading to conditions like bronchitis and asthma [23]. PM10 and PM2.5 particles enter the respiratory system through inhalation via the mouth or nose [75]. They deposit in specific regions of the lungs, penetrate, or are absorbed by the mucous layer, causing localized damage. PM10 can penetrate the upper respiratory tract and deposit through impaction or sedimentation processes; PM2.5 particles, on the other hand, deposit primarily in the lungs, especially in the alveoli, and can transfer to various organs through systemic circulation from the lungs [76]. Inflammation in the respiratory system and disruptions in the microbiome balance can extend beyond local areas, potentially affecting the mouth and even the brain via the “oral–lung axis,” increasing the susceptibility to oral and neurological disorders [77, 78]. Additionally, respiratory inflammation can impact intestinal health through the “lung–gut axis.” Studies indicate that lung infections and inflammation can disrupt the gut bacterial balance and damage the intestinal lining through various pathways, including the connection between the lungs and the intestines and interactions between bacteria and the gut. This may have a role in the beginning and growth of intestinal diseases [79,80,81,82,83]. Concurrently, an imbalance in the intestinal flora can exacerbate respiratory tract inflammation through the gut–lung axis, creating a vicious cycle [84].

The gastrointestinal system acts as another critical point of contact for the body. Airborne toxins, especially PM2.5, may travel through the lungs, where they are engulfed by alveolar macrophages and cleared by the mucociliary system, ultimately being swallowed into the gastrointestinal tract via the oropharynx or accumulate in the gut by consuming contaminated food and water, directly harming gut bacteria and the protective intestinal lining [80, 85,86,87,88]. Research has shown that imbalances within gut bacteria perform an important part in the development of cardiovascular and endocrine disorders stemming from exposure to air pollution [89, 90]. Disruption of intestinal flora can influence brain function through various mechanisms, including afferent channels of the vagus nerve, enterogenic inflammatory factors entering brain tissue, and endogenous hormone mediators [91,92,93]. Research has demonstrated that antibiotics can regulate the gut microbiota, resulting in reduced neuroinflammation and cognitive decline in mice exposed to PM2.5, highlighting the substantial impact of gut microbiota upon the neurological impacts of air pollution [94]. Furthermore, disturbances within intestinal flora can also exacerbate respiratory tract inflammation through the “gut–lung axis,” while lung infections and inflammation can worsen disturbances in intestinal flora and damage to the intestinal barrier through the “lung–gut axis, creating a vicious cycle [95, 96].

Air pollution can directly harm the central nervous system in several ways, in addition to their indirect effects on brain function through the oral–entero–brain axis network. PM2.5 and other harmful substances in the air can penetrate the olfactory bulb via the olfactory epithelium and travel to the brain through the olfactory pathway, causing inflammation in the nervous system and damaging nerve cells [97,98,99]. Research has found that particles from burning fuel (CDNPs) occur within the brains of young individuals in Mexico City, demonstrating that airborne pollutants can reach the brain and damage neurovascular units from an early age [100]. Furthermore, the systemic inflammation caused by inhaled pollutants can facilitate the entry of peripheral inflammatory agents into brain tissue by compromising the blood–brain barrier (BBB) and exacerbate neuroinflammation [101]. Airborne toxic substances may also trigger the death of neuronal cells via stimulating microglia and astrocytes, leading to disruptions in dopamine, acetylcholine, and other neurotransmitters and impairing cognitive, movement, and other nervous system functions [102, 103]. The disruption of oral and intestinal flora can also impact brain health through the oral–olfactory–brain axis and entero–brain axis [77]. In summary, the lung, intestine, and brain are tightly interconnected through the oral–entero–brain axis network, with local injuries having the potential to rapidly spread throughout the body.

**Figure 1.**
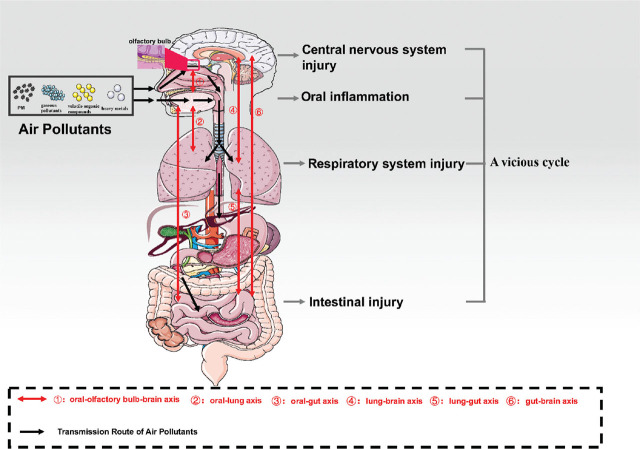
The transmission route of air pollution components in the oral–gut–brain axis.

Overall, we are pleased to find that the impacts of air pollutants on the oral–gut–brain connection are not unilateral but interact and exacerbate each other. Small molecular air pollutants increase their absorption and accumulation in the body by damaging the mucosal barrier. Simultaneously, they disrupt the overall balance of flora by altering local flora disturbances and inducing inflammation that evolves from local to systemic. The most harmful aspect of air pollution is its damage to the nervous system, but neuroendocrine dysfunction further aggravates local and systemic pathological changes. Thus, the oral–enterobrain axis injury induced by air pollutants forms a vicious cycle of “pollution—inflammation—flora disturbance—nerve damage” (**Figure 1**).

## Related molecular mechanisms of air pollution components to body injury

Airborne toxins can harm the body by impacting the in vivo connection between the mouth, gut, and brain through various biological processes, resulting in health and longevity issues. These substances can cause inflammation and oxidative stress, activating pathways like nuclear factor kappa B (NF-κB), which increases the release of inflammatory substances and reactive oxygen species (ROS), damaging cells and tissues. Additionally, air pollutants can induce epigenetic variations, which include abnormal DNA methylation (DNAm), alterations in histone structure, and fluctuations in non-coding ribonucleic acid (RNA) levels, which disturb gene transcription and protein synthesis, ultimately disrupting cellular internal balance. It is essential to investigate the molecular processes triggered via air pollutants and their operational modes within the oral–entero–brain connection to understand the molecular foundations of diseases caused by air pollution **(Table 2)**.

**Table 2.** Related molecular mechanisms of air pollution components to body injury

**Categories**		**Targets**	**Reference**
Inflammatory response	Pulmonary inflammatory response	MAPK/AP-1, JAK/STAT, NF-κB activation; NLRP3 inflammasome#; TNF-α, IL-1β, IL-6#	[105,106,107,108,109,110]
	Intestinal inflammatory reaction	MMP-9 and TLR4#, Tight junction protein$; Increased intestinal permeability	[86, 111]
	Neuroinflammatory response	The integrity of the BBB destroyed; microglia and astrocytes activated IL-1β#; oAβ# Vitamin B12 metabolism imbalance, neurotoxicity	[112, 113] [114] [115]
Oxidative stress		ROS# MAPK pathway activated ICAM-1#, VCAM-1#, MMPs activated	[114] [116] [117, 118]
Mitochondrial dysfunction		Overproduction of ROS, NOX4/Nrf2 REDOX imbalance, mitochondrial membrane potential reduced Bcl-2#, Bax#, caspase# mTOR activated, LC3#, beclin1# PINK1/parking activated	[119] [120, 121] [122, 123] [124]
Epigenetic changes	DNAm	FOXP3 oral cell promoter methylation Hypomethylation of genomic DNA in lung tissue and abnormal DNAm of IL-4 and IFN-γ ROS-Akt-DNMT3B hypermethylation	[125] [126] [127]
	Histone modification	H3K27me3 $ HDAC2# HDAC2#, Sirtuin 6# H3K9 acetylation# H3K9me2/me3 $, γ-H2A.X #	[128, 129] [130] [131] [132] [97]
	Non-coding RNA	miR-6238, miR-146, miR-139, miR-340# miR-155, lncRNA SOX2-OT ceRNA lncRNA NEAT1# TGF-βR2/miR-466h-3p-IL-17Rα/Aβ-42/Ache and miR-125b-Pcdhgb8 miR-574-5p miR-6315, miR-3588, miR-466b-5p, miR-3560, let-7b-5p#, miR-99b-5p, miR-92b-5p, miR-99a-5p, miR-338-5p, let-7e-5p $	[133, 134] [135] [136] [137]

BBB, blood-brain barrier; DNAm, DNA methylation; HDAC2, histone deacetylase 2; IFN-γ, interferon-γ; lncRNA, Long-chain non-coding RNAs; JAK/STAT, Janus kinase/signal transducer and activator of transcription; MAPK/AP-1, mitogen-activated protein kinase/activator protein 1; MMP-9, Matrix metalloproteinase-9; NLRs, NOD-like receptors; NF-κB, nuclear factor kappa B; PINK1, PTEN induced putative kinase 1; ROS, reactive oxygen species; SIRT6, Sirtuin 6; TNF-α, tumor necrosis factor alpha.

## Inflammatory response

The health impacts of air pollutants are profoundly influenced by inflammatory responses. Pollutants like PM2.5 and PM10 can increase the presence of bacteria, fungi, viruses, and other microorganisms, along with their byproducts recognized as pathogen-associated molecular patterns (PAMPs) like lipopolysaccharide (LPS), peptidoglycan (PGN), as well as flagellin [104]. These can activate both innate and adaptive immunity in various ways, causing a systemic inflammatory response. Airborne toxins have the capacity to engage specific pattern-recognition receptors, which include NOD-like receptors (NLRs) and Toll-like receptors (TLRs). They can also activate critical transcription factors like Janus kinase/signal transducer and activator of transcription (JAK/STAT), mitogen-activated protein kinase/activator protein 1 (MAPK/AP-1), and NF-κB. Additionally, they can stimulate the NLR family, specifically the pyrin domain containing 3 (NLRP3) inflammasome, promoting the release of tumor necrosis factor alpha (TNF-α), interleukin-1beta (IL-1β), interleukin-6 (IL-6), and other inflammatory molecules, leading to both local and systemic inflammation [105,106,107,108,109,110].

In addition, air pollutants also influence intestinal inflammatory responses in various ways. Research has shown exposure to PM2.5 in mice findings within elevated levels of Matrix metalloproteinase-9 (MMP-9) and TLR4, stimulation of the NF-κB signaling pathway, a reduction in tight junction protein expression, and increased intestinal permeability [86, 111]. The influx of PAMPs triggers a pro-inflammatory response in macrophages and dendritic cells, leading to systemic inflammation, further deteriorating intestinal permeability and exacerbating the lumen environment of the intestine [20]. PM2.5 also significantly affects the central nervous system. Different mechanisms can impact brain function, such as compromising the BBB, stimulating microglia and astrocytes, which includes neuroinflammation, and disrupting neurotransmitter production and release, which leads to impaired neuronal function [112,113,114]. Research has revealed that exposure to PM2.5 exacerbates neuronal damage and inflammation driven by oligo-amyloid beta (OA-β) within neuron-microglia co-cultures via increasing IL-1β production [115]. PM2.5 exposure causes neurotoxicity by disrupting the gut microbiota and disturbing vitamin B12 metabolism [116]. It causes a neuroinflammatory response and cognitive impairment, with the olfactory bulb and hypothalamus experiencing inflammation first [117]. Multiple studies indicate that air pollutants like PM2.5 could pose a significant environmental risk for neuroinflammatory dementia [118].

Interestingly, when the body is continuously exposed to high concentrations of PM2.5, it exhibits a damage–repair-imbalance response pattern [119]. Studies have discovered that during the first 4 weeks of exposure, mice display depressive behavior, accompanied by a persistent upregulation of pro-inflammatory cytokines and chemokines, indicating that PM2.5 exposure causes significant harm to the organism [120]. However, when PM2.5 exposure is discontinued, these pathophysiological changes return to normal levels within 2 weeks, suggesting that the body possesses a certain degree of self-repair capacity. This enables the body to independently repair the damage induced by PM2.5 after exposure termination. For example, type 2 alveolar epithelial cells initiate rapid repair of the alveolar epithelial mucosa through proliferation [121]. It is noteworthy that when mice previously exposed to PM2.5 are re-exposed to the same dose of PM2.5, their target organs demonstrate increased sensitivity, implying that prior exposure may have already caused some level of persistent damage to the body, making it more susceptible to the effects of subsequent PM2.5 exposure [122, 123].

## Oxidative strain and malfunctioning mitochondria

One important way by which air contaminants cause harm is oxidative stress. Air pollutants can directly produce ROS or stimulate endogenous ROS production through enzymes like NADPH oxidase (NOX), nitric oxide synthase (NOS), and cyclooxygenase, leading to oxidative overreactions and antioxidant imbalances in the body [124]. In OA-β-stimulated microglia, PM2.5 exposure has been displayed to increase ROS levels [115]. Additionally, an excess of ROS induced through PM2.5 activates MAPK pathways and transcription variables like NF-κB and AP-1, which results in increased production of inflammatory proteins [125]. Furthermore, an overabundance of ROS can also promote the infiltration of inflammatory cells, activate MMPs, degrade the extracellular matrix, impair epithelial barrier function, and increase the permeability of endothelial blood vessels by upregulating the expression of intracellular adhesion molecules such as intercellular adhesion molecule-1 (ICAM-1) and vascular cell adhesion molecule-1 (VCAM-1) [126,127,128,129,130].

Mitochondrial dysfunction has a major impact upon the health effects of air pollution. Various mechanisms, such as DNA damage, decline in membrane potential, and overexpression of ROS, can lead to mitochondrial damage induced by air pollutants. This can cause disruptions in energy metabolism and cell apoptosis [131,132,133,134]. Low exposure levels to PM2.5 (200 μg/mL) increase ROS production, exacerbate imbalances in NOX4/Nrf2 Reduction-Oxidation (Redox), reduce mitochondrial membrane potential, and ultimately intensify oxidative stress as well as mitochondrial damage [135]. PM2.5 exposure elevates ROS levels, apoptosis, and the expression of apoptosis-related proteins within mouse brain tissue, indicating that mitochondrial dysfunction contributes to the neurotoxicity of air pollutants [136]. Additionally, air pollutants can activate both intrinsic and extrinsic pathways of cell death by regulating proteins connected to cell death like b-cell lymphoma-2 (Bcl-2), Bcl-2 associated X protein (Bax), and caspase, leading to cell apoptosis [137,138,139]. Exposure to PM2.5 exacerbates behavioral abnormalities associated with Parkinson's disease by increasing oxidative stress along with triggering mitochondria-mediated neuronal cell death [140]. Fine PM exposure results in oxidative damage that can disrupt cellular energy production and lead to apoptosis [141]. Autophagy, an essential cellular process for removing damaged organelles and maintaining cellular equilibrium, has been shown to provide a temporary defense against PM2.5-induced cell death in some studies [142]. However, it is noteworthy that other research has suggested that exposure to PM2.5 can inhibit the M2 polarization of anti-inflammatory macrophages via the mammalian target of rapamycin (mTOR)-dependent pathway, and promote the overexpression of autophagy-related genes *LC3* and *beclin1*, leading to increased autophagy and neuronal death [143,144,145]. PM2.5 exposure may also induce mitochondrial autophagy and organ fibrosis by raising ROS levels and activating the PTEN-induced putative kinase 1 (PINK1)/Parkin signaling pathway [146]. Autophagy and apoptosis interactively mediate the cytotoxic effects of air pollutants.

## Epigenetic changes

Environmental factors influence health through significant changes in epigenetic processes. Air pollutants regulate inflammation, apoptosis, oxidative stress, and other pathological processes through various epigenetic mechanisms such as DNAm, histone modification, and non-coding RNA.

Air pollutants are key in causing injury in the oral–gut–brain axis by altering DNAm patterns and affecting downstream gene expression. Studies have observed fluctuating short-term variations within DNAm and hydroxymethylation levels about oral cells, displaying a negative correlation alongside exposure duration to PM2.5 and PM10 [147]. Urban children exposed to high levels of air pollutant black carbon (BC) might exhibit lower FOXP3 oral cell promoter methylation during physical activity, potentially indicating improved T regulation (Treg) function and improved lung function [148]. Additionally, investigations into the respiratory system have displayed that exposure to PM2.5 leads to reduced methylation of DNA in mouse lung tissue and abnormal methylation of IL-4 and interferon-γ (IFN-γ), resulting in lung damage and pathological alterations [149]. Following PM2.5 exposure, Nrf2 may trigger DNA demethylation via ten–eleven translocation 3 (TET3) expression, exacerbating lung inflammation [150]. Exposure to PM2.5 can increase the likelihood of lung cancer by promoting systemic inflammation and decreasing DNAm in white blood cells, potentially due to PM2.5 exposure inhibiting *P53* gene activity by increasing methylation in its promoter region through the ROS-protein kinase B (Akt)-DNA methyltransferase 3B (DNMT3B) pathway [151]. Research found altered methylation levels in 4 out of 15 cancer-related loci, such as HPP1, Cdh1, GDNF, and MYOD1, in individuals having active ulcerative colitis in contrast to those having healthy mucosa [152]. By contrast, genes like *CXCL5*, *ATF2*, and *CXCL6*, along with *interleukin 12A (IL12A)*, *inhibin alpha (INHA)*, *IL15*, *IL12B*, *CXCL3*, *interleukin 17 receptor A (IL17RA)*, *interleukin-6 receptor (IL6R)*, *IL6ST*, *FADD*, *CCL25*, *CXCL14*, *IL4R*, *GATA3*, *IL7*, and tyrosine kinase 2 (*TYK2)*, showed hypomethylation in Crohn's patients relative to healthy controls [153, 154]. However, these findings are based primarily on simple DNAm correlation studies, and unfortunately, research on DNAm in the body brought on by exposure to air pollutants is scarce. Notably, abnormal DNAm is also strongly linked to air pollution-related neurological disorders. Research has shown that varying DNAm related to brain inflammation facilitates the connection between PM2.5 and AD [155].

Histone modification is another critical epigenetic regulatory mechanism. Research has shown that exposure to pollutants can mediate the upregulation of CXCL2 in gum mesenchymal stem cells (GMSCs) and periodontal ligament stem cells (PDLSCs), reduce H3K27me3, activate distal enhancers, and exacerbate oxidative stress damage in the oral mucosa [156, 157]. Exposure to fine PM, in combination alongside a high-fat diet, can induce lung damage by altering various histone modifications linked with DNA damage [158]. PM2.5 promotes M2 polarization by inhibiting histone deacetylase 2 (HDAC2), which can lead to chronic obstructive pulmonary disease [159]. In mice, HDAC3 exacerbates lung harm resulting from PM2.5 through regulation of the transforming growth factor beta (TGF-β)/Smad2/3 and NF-κB pathways [160]. Sirtuin 6 (SIRT6), a type III histone deacetylase, increases airway inflammation in macrophages due to PM2.5 [160]. Persistent exposure to air contaminants significantly increases H3K9 acetylation levels in peripheral blood mononuclear cells (PBMCs) and lung tissue [161]. Neurological disorders are associated with abnormal histone changes due to air pollution. Studies involving residents or mice exposed to particulate air pollution have shown decreased H3K9me2/me3, increased γ-H2A.X staining, and elevated levels of tau phosphorylation linked with AD [97].

Long-chain non-coding RNAs (lncRNA) and microRNA (miRNA) are also key factors in the impacts of air pollution on oral–gut–brain axis abnormalities. Research has shown that exposure to PM2.5 significantly increases levels of miR-139, miR-6238, miR-146, and miR-340 in lung tissue, exacerbating inflammatory reactions and oxidative stress [162, 163]. Moreover, miR-146 may act as a novel regulator that promotes inflammatory responses through its interactions with the NF-κB signaling pathway [164]. Additionally, miR-155, lncRNA SRY-box transcription factor 2 overlapping transcript (SOX2-OT) ceRNA, and lncRNA NEAT1 have been shown to increase the aggressiveness of human bronchial epithelial cells under prolonged exposure to PM2.5 [165, 166]. Thirteen miRNAs connected to lung cancer have been identified within both mouse serum and lung tissue, indicating their function within the progression and development for the disease. Ning et al. [167] identified various miRNA target genes, including *ATG10*, *BAX*, *E2F1*, *CDK6*, *MAP3K7*, *CTNNB1*, *UBE2V2*, *CRK*, *NR2F2*, *VIM*, *HIF1A*, *CCND2*, *PRKCA*, *SIRT1*, *RASSF5*, and *RASSF1*, among 13 other unregulated genes. Air pollution can also alter miRNA expression in the intestines, specifically targeting the suppression of ZO-1 tight junction protein expression and impairing intestinal barrier function [86]. miRNA and long non-coding RNA significantly contribute to nerve damage caused by exposure to air pollutants. Research has displayed that miRNAs from the let-7a group (mmu-let-7a) can induce changes in mouse cerebrovascular endothelial cells (mCEC) after inhaling fine PM [168]. Exposure to PM2.5 has exacerbated cognitive and structural brain damage in mice with AD, leading to elevated levels of inflammatory cytokines (TNF-α and IL-6), AChE, and Aβ-42, and decreased ChAT levels. This study highlighted a strong correlation between brain injury and two sets of miRNA target genes: transforming growth factor beta receptor 2 (TGF-βR2)/miR-466h-3p-IL-17Rα/Aβ-42/Ache and miR-125b-Pcdhgb8 [169]. Inhalation of PM2.5 can cause neuroinflammation, damage synaptic function, and impair spatial learning and memory, with these impacts connected to the activation of BACE1 through the suppression of miR-574-5p controlled by NF-κB p65 [170]. PM2.5 exposure alters miRNA expression within the brain, affecting the cortex (miR-99b-5p, miR-92b-5p, miR-466b-5p, miR-99a-5p, miR-6315, miR-3588, miR-338-5p) and hippocampus (let-7b-5p, miR-3560, let-7e-5p), linked to genes associated with astrocyte migration (Pkn2), neurite outgrowth (Gorab), and allergic encephalomyelitis (Mobp), ultimately influencing cognitive development and physical coordination [171].

## Summary and prospect

**Figure 2.**
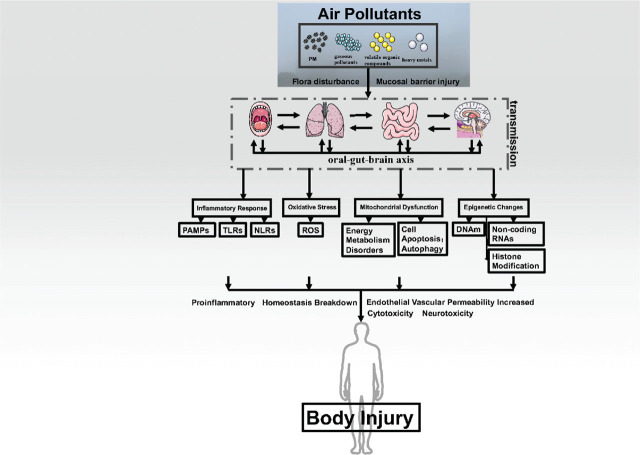
The correlative pathway of air pollutants to body injury. NLRs, NOD-like receptors; PAMPs, pathogen-associated molecular patterns; ROS, reactive oxygen species; TLRs, Toll-like receptors.

In conclusion, the interconnectedness of the oral, gut, and brain, recognized as the oral–gut–brain axis, is crucial for understanding how air pollutants and their consequent effects influence our lives, health, and economic assets. Inhalation of air pollutants results in degradation of the mucosal barrier, disturbances in bacterial flora, and inflammatory responses in the mouth and the respiratory and digestive tracts. These pollutants also interact through the network that connects the oral, intestinal, and brain systems, causing damage to various organs and systems in the body. Air pollutants induce abnormalities in the oral–gut–brain axis through inflammatory responses, oxidative stress, mitochondrial dysfunction, and epigenetic changes (**Figure 2**). This study deepens our understanding of how air pollutants impact the oral–gut–brain connection and explores how various pollutants collaborate within this connection. These critical findings will provide a solid scientific foundation for future precise assessments of the risks associated with air pollution exposure to life and health. Additionally, they will aid governments and academic communities in developing specific preventive measures and control strategies.
